# Metabolomic responses are more sensitive in muscle than serum following 28 days of arduous exercise with erythropoietin administration

**DOI:** 10.1113/EP093342

**Published:** 2026-04-11

**Authors:** Devin J. Drummer, Emily E. Howard, Julie L. McNiff, Marques A. Wilson, Christopher T. Carrigan, Nancy E. Murphy, Jess A. Gwin, David E. Barney, Benjamin J. Ryan, James P. McClung, Stefan M. Pasiakos, Lee M. Margolis

**Affiliations:** ^1^ Military Nutrition Division U.S. Army Research Institute of Environmental Medicine Natick Massachusetts USA; ^2^ Oak Ridge Institute for Science and Education Belcamp Maryland USA; ^3^ Combat Feeding Division U.S. Army Combat Capabilities Development Command (DEVCOM) Natick Massachusetts USA; ^4^ Thermal and Mountain Medicine Division U.S. Army Research Institute of Environmental Medicine Natick Massachusetts USA; ^5^ Military Performance Division U.S. Army Research Institute of Environmental Medicine Natick Massachusetts USA; ^6^ Center for Human Performance Optimization Penningtion Biomedical Research Center Baton Rouge Louisiana USA

**Keywords:** aerobic exercise, network biology, substrate utilization

## Abstract

Erythropoietin (EPO) administration stimulates haematological and non‐haematological adaptations that alter substrate oxidation and enhance aerobic performance. The effects of strenuous exercise and EPO on metabolites, and whether any effect is associated with haematological and non‐haematological adaptations, has not been assessed. The objective of this study was to examine changes in serum and skeletal muscle metabolomes and explore whether changes were associated with haematological and non‐haematological adaptations to strenuous exercise and EPO. Eight males (20 ± 3 years, 25 ± 3 kg/m^2^) completed this longitudinal study. Participants received 50 IU/kg body mass of EPO, 3×/week for 28 days, while exercise (energy expenditure of 1200–1500 kcals/day) and diet were controlled. Before (PRE) and after (POST) EPO, ${\dot{V}}_{O_{2}\mathrm{peak}}$, time trial (TT) performance, blood volume, iron homeostasis, and untargeted metabolomics profiles in rested/fasted muscle and serum were assessed. Weighted gene correlation network analysis (WGCNA) was used to identify plasticity of metabolite networks (POST/PRE) correlated with change in ${\dot{V}}_{O_{2}\mathrm{peak}}$, TT performance, blood volume and iron homeostasis. Four serum and 51 muscle metabolites were different (*P* ≤ 0.05, false discovery rate ≤ 0.10) at POST compared to PRE. Of the WGCNA networks identified, two serum modules were associated with aerobic performance (*P* = 0.05), while five skeletal muscle modules were associated with both aerobic performance and iron outcomes (*P *= 0.05). Changes to the serum and skeletal muscle metabolomes indicate altered carnitine, β‐alanine and glucose metabolism metabolites, likely depicting a shift to greater fat oxidation, buffering capacity and the alteration in iron homeostasis following 28 days of EPO administration and strenuous exercise.

## Abstract (highlights)

- **What is the central question of this study?**
  What changes in serum and skeletal muscle metabolomes occur and are they associated with haematological and non‐haematological adaptations to strenuous exercise and exogenous erythropoietin (EPO) administration?
- **What is the main finding and its importance?**
  The skeletal muscle metabolome was more sensitive to 4 weeks of strenuous exercise and exogenous EPO administration than serum, and changes in carnitine, β‐alanine and glucose metabolism metabolites likely depict a shift to greater fat oxidation, buffering capacity and the alteration in iron homeostasis following 28 days of exogenous EPO administration and strenuous exercise training.

## 1 INTRODUCTION

Erythropoietin (EPO) administration alone and coupled with exercise is a potent stimulator of erythropoiesis, resulting in robust haematological adaptations such as increased red cell volume (Lundby & Olsen, 2011; Lundby et al., 2008; Ryan et al., 2024), haemoglobin and haematocrit (Drummer et al., 2024; Durussel et al., 2013; Thomsen et al., 2007) that contribute to increased oxygen carrying capacity and ${\dot{V}}_{O_{2}\mathrm{peak}}$ (Drummer et al., 2024). Exogenous EPO also stimulates non‐haematological adaptations (Annaheim et al., 2016), leading to metabolic alterations and shifts in substrate oxidation (Yin et al., 2024). Specifically, EPO administration increases fat oxidation (Caillaud et al., 2015; Christensen et al., 2012; Drummer et al., 2024; Larsen et al., 2021), likely due to enhanced mitochondrial biogenesis and respiratory capacity (Drummer et al., 2024; Guadalupe‐Grau et al., 2015; Larsen et al., 2021; Plenge et al., 2012; Wang et al., 2013). When strenuous exercise and EPO administration are performed together, the reliance on carbohydrate as a fuel source is reduced and the metabolic clearance rate of glucose from circulation is enhanced (Caillaud et al., 2015; Drummer et al., 2024). Ultimately, enhancements in oxygen carrying capacity and shifts in substrate oxidation from strenuous exercise and EPO result in physical performance improvements (Drummer et al., 2024). The mechanisms underpinning these adaptations remain unclear.

High‐throughput global metabolomics analysis may provide a comprehensive understanding of underlying metabolic adaptations facilitating alterations in iron homeostasis, substrate oxidation and physical performance with EPO (Marciano & Snyder, 2019). In a recent study, participants were administered 50 IU/kg EPO twice per week for 28 days, and changes in metabolomics profiles within plasma, serum and urine were observed (Lima et al., 2023). This work captured temporal changes in the metabolome across each biospecimen, with some metabolite shifts correlating with haematological parameters (Lima et al., 2023). These findings provide insight into the mechanisms driving EPO adaptions in the most commonly collected biospecimens for metabolomics (Giskeødegård et al., 2019), but other tissues such as muscle have not been examined. Assessment of metabolites in muscle may provide context for underlying tissue metabolism (Graham et al., 2019), and contribute to the understanding of non‐haematological adaptations.

The objective of this study was to examine changes in serum and skeletal muscle metabolomics and explore if these changes are associated with the haematological and non‐haematological adaptations to strenuous exercise and EPO. In addition to determining changes in these two metabolomes, this work sought to leverage correlation techniques (weighted correlation network analysis; WGCNA) to understand which clusters of metabolites drive changes in ${\dot{V}}_{O_{2}\mathrm{peak}}$, time trial (TT) performance and iron homeostasis. We hypothesized a similar abundance of differential metabolites between serum and skeletal muscle in the rested and fasted state, with each metabolome exhibiting hubs linked to performance, haematological status and iron regulation.

## 2 METHODS

### 2.1 Ethical approval

The study followed the ethical standards of the *Declaration of Helsinki*. The study was registered on clinicaltrials.gov (NCT05078138). All study procedures were approved by the U.S. Army Medical Research and Development Command (Fort Detrick, Fredericksburg, MD, USA) Institutional Review Board (IRB#: M‐10894). Each participant provided written informed consent prior to beginning data collection.

### 2.2 Participants

This study is part of a larger investigation (Drummer et al., 2024; Ryan et al., 2024) that assessed haematological and non‐haematological adaptations to 28 days of exogenous EPO administration with strenuous exercise training. In brief, eight healthy males (age: 20 ± 3 years, height: 1.77 ± 0.03 m, body mass: 78.91 ± 8.68 kg, body mass index: 25.17 ± 3.10 kg/m^2^, ${\dot{V}}_{O_{2}\mathrm{peak}}$: 44.13 ± 2.39 mL/kg/min) completed this longitudinal study at the U.S. Army Research Institute of Environmental Medicine. Eligible participants were males or females who were recreationally active (2–4 days per week of aerobic and/or resistance exercise) and weight stable (±2.3 kgs) for at least 2 months prior to the onset of the study. All participants were US Army service members and free from known acute or chronic diseases (e.g., cardiovascular, pulmonary and metabolic disease), food allergies and medication allergies at screening. Participants were required to refrain from consuming alcohol or nicotine products for the duration of the study.

### 2.3 Study design

**FIGURE 1:**
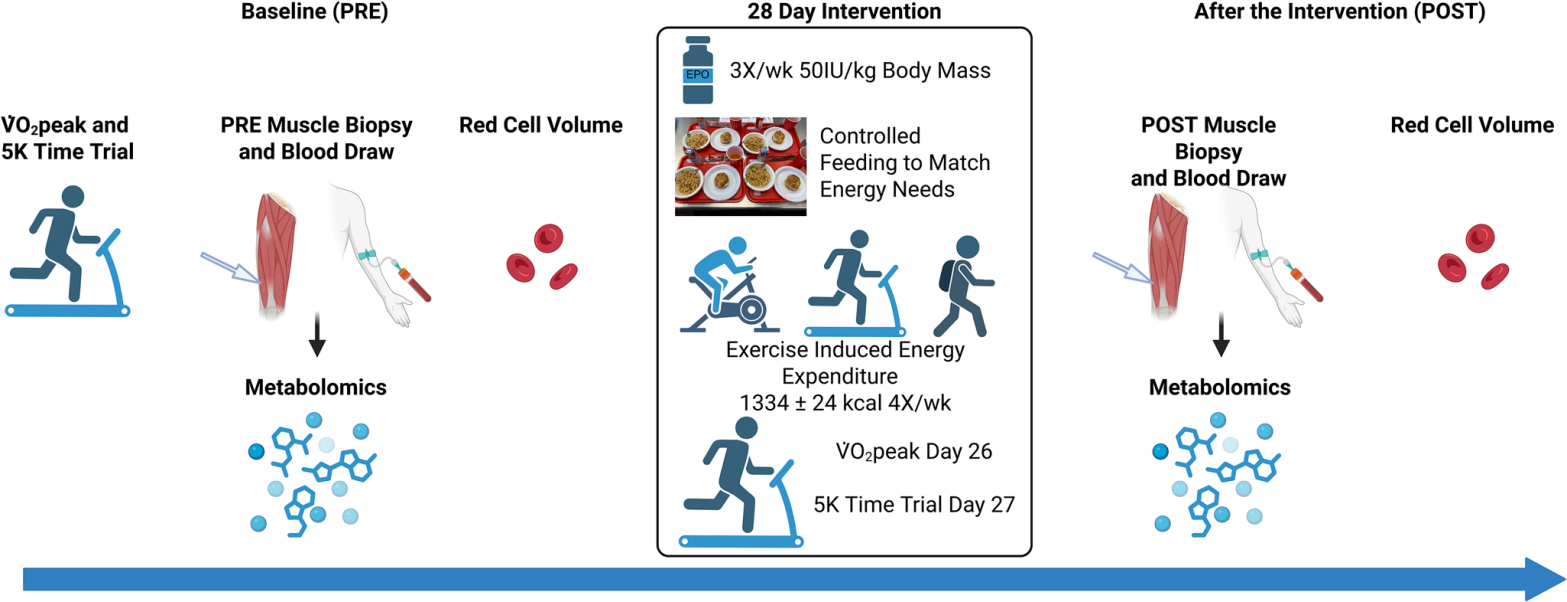
Study design depicting the collections at baseline (PRE) and after 28 days of EPO and controlled exercise and feeding (POST).

This longitudinal study consisted of a baseline phase, followed by 28 days of EPO injections (50 IU/kg body mass subcutaneously 3×/week; PROCRIT, EPOETIN ALFA, Janssen Products, LP, Titusville, NJ, USA) while controlling exercise and dietary intake (Figure 1). Assessments of ${\dot{V}}_{O_{2}\mathrm{peak}}$, aerobic performance (5 km TT), red cell volume, markers of circulating and skeletal muscle iron status, and serum and skeletal muscle metabolomics were completed in a rested fasted state at baseline (PRE) and after (POST) the final administration of EPO (day 26). During the 28‐day intervention, participants exercised 5×/week. Exercise training was controlled and consisted of a prescribed combination of endurance‐ and resistance‐type exercise (Drummer et al., 2024). Exercise‐induced energy expenditure was 1215 ± 56 kcal/day for the first 2 weeks, and 1453 ± 56 kcals/day for the second 2 weeks of the intervention (Drummer et al., 2024).

Registered dietitians developed individualized daily menus using Food Processor SQL (ESHA Research, Salem, OR, USA; Version 10.14). Energy content of diets was prescribed to match each participant's estimated energy needs to maintain body weight throughout the study. Dietary iron was controlled throughout the study. All food and beverages (except water) were provided to participants for the duration of the study. Water consumption was ad libitum. Average daily dietary intake throughout the intervention was 3162 ± 265 kcal/day; 5.6 ± 0.2 g carbohydrate/kg/day; 1.6 ± 0.1 g protein/kg/day; 1.3 ± 0.1 g fat/kg/day; and 39.5 ± 4.4 mg iron/day (Drummer et al., 2024). Body weight and composition remained consistent from PRE to POST (weight PRE = 78.9 ± 8.7 kg, POST = 78.5 ± 9.0 kg; fat mass PRE = 18.1 kg, POST = 17.6 kg) (Drummer et al., 2024).

### 2.4 ${\dot{V}}_{O_{2}\mathrm{peak}}$

Participants completed a maximal aerobic exercise test to determine peak oxygen uptake (${\dot{V}}_{O_{2}\mathrm{peak}}$) on a treadmill following a 10‐h overnight fast (Drummer et al., 2024). ${\dot{V}}_{O_{2}\mathrm{peak}}$ was determined using an indirect, open circuit respirator system (Parvomedics, Sandy, UT, USA) at PRE and day 26 of the intervention. Participants completed a 3‐min self‐paced warm‐up on the treadmill, then began running for 4 min at a pace predetermined as comfortable at a 0% grade. At 4 min, the grade was increased to 4% followed by an additional 2% every 2 min thereafter until volitional exhaustion.

### 2.5 TT performance

Following a 10‐h overnight fast, participants completed a 5 km TT on day 27. The treadmill was set at a constant 1% grade for the entire test to accurately mirror outdoor running (Jones & Doust, 1996). Following a warm‐up period, the treadmill was set to 5 mph (8 km/h), then participants adjusted the treadmill speed to complete the distance as quickly as possible. Participants were blinded to the treadmill speed. No motivation was provided during the TT. The only feedback given was distance covered at half mile increments. Participants were allowed to consume water *ad libitum* throughout. Participants completed a minimum of two practice exercise sessions to ensure they were familiar with the performance test prior to the first TT of record.

### 2.6 Plasma and muscle iron homeostasis

The optimized carbon monoxide rebreathing method (Schmidt & Prommer, 2005) was used to assess red cell volume at baseline, day 29 and day 30. In brief, carboxyhaemoglobin was measured in quintuplicate (OSM3 Hemoximeter, Radiometer, Copenhagen, Denmark) from a prewarmed finger before and 5 min after 2 min of rebreathing a small bolus of carbon monoxide to determine total haemoglobin mass (Ryan et al., 2024). Red cell volume was calculated using haemoglobin mass as previously described (Schmidt & Prommer, 2005). Plasma ferritin and soluble transferrin receptor (sTfr) concentrations were assessed using the Access 2 Immunoassay System (Beckman Coulter, Brea, CA, USA) as previously described (Ryan et al., 2024). Samples were collected after a 10‐h overnight fast on day 28. All measurements were assessed in duplicate. Intraassay coefficients of variation were 2% for red cell volume, ferritin and sTfr.

A resting and fasted skeletal muscle biopsy was collected from the vastus lateralis PRE and POST. Muscle ferritin, transferrin receptor and myoglobin (Mgb) protein abundances were determined via immunoblotting, using methods previously detailed (Ryan et al., 2024). Briefly, protein from each sample was separated using SDS‐PAGE and transferred onto polyvinylidene difluoride membranes. Membranes were then blocked and incubated overnight at 4°C with appropriate primary antibodies for ferritin (ab75973; Abcam, Waltham, MA, USA; RRID:AB_1310222; 1:1,000), transferrin receptor (No. 13113; Cell Signaling Technology, Danvers, MA, USA; RRID:AB_2715594; 1:1,000) and Mgb (No. 25919; Cell Signaling Technology; RRID:AB_2798916; 1:2,000). After being washed, membranes were incubated with secondary antibodies and imaged (ChemiDoc XRS, Bio‐Rad Laboratories, Hercules, CA, USA). The abundance of ferritin, Mgb and transferrin receptor were normalized to the abundance of heat shock protein‐90 (HSP90; reported previously; Drummer et al., 2024; Ryan et al., 2024) and POST was expressed as a ratio to mean relative abundance at PRE.

### 2.7 Metabolomics

Serum (*n* = 7 paired; 14 total) and skeletal muscle (*n* = 8 paired; 16 total) samples for metabolomics were collected under resting, fasted conditions. Samples were shipped to Metabolon Inc., Morrisville, NC, USA and stored at −80°C until processed using Metabolon's workflow. Briefly, samples were prepared with the MicroLab STAR® system (Hamilton Company, Reno, NV, USA) and standards added prior to extraction for quality control (QC). Proteins were precipitated using methanol and shaking then centrifuged. TurboVap® (Zymark) was briefly used to remove organic solvent from samples. A pooled sample consisting of the combination of experimental samples served as a technical replicate while water served as blanks, and QC standards were spiked into each sample. Samples were analysed using four separate methods: two separate reverse‐phase (RP)/ultra‐performance liquid chromatography (UPLC)–tandem mass spectrometry (MS/MS) methods with positive ion mode electrospray ionization (ESI), a RP/UPLC‐MS/MS method with negative ion mode ESI, and a hydrophilic interaction liquid chromatography (HILIC)/UPLC‐MS/MS method with negative ion mode ESI.

Raw data were extracted, peaks identified, and quality control processed using proprietary hardware and software (serum median relative standard deviation (RSD): internal standards = 5%, endogenous biochemicals = 8%; muscle median RSD: internal standards = 3%, endogenous biochemicals = 7%).

### 2.8 Statistical and bioinformatics analysis

Metabolomic peak intensities were uploaded to MetaboAnalyst 6.0 (Pang et al., 2024) (serum: 1347 metabolites with 1098 metabolites of known identity; muscle: 755 metabolites with 682 metabolites of known identity). Features with >20% missing values were removed with all remaining missing values imputed with 1/5 the minimum value. Variance filters were employed using default empirical rules (interquartile range (IQR) of 40% for serum and 25% for muscle) based on the number of variables (655 and 495 metabolites for serum and muscle remained respectively). Principal component analyses for serum and muscle are shown in Supporting information, Figure S1. Data were log10 transformed and analysed for differential abundance between PRE and POST (Student's paired *t*‐test). Significant thresholds were determined as *P* ≤ 0.05 and false discovery rate (FDR) ≤ 0.10 limiting the false positives to 10% in these exploratory analyses. Weighted gene correlation network analysis (WGCNA) (Langfelder & Horvath, 2008, 2012) was performed in R (version 4.4.2) to find metabolite networks and determine associations with relevant outcomes as previously described (Lavin et al., 2020, 2021; O'Bryan et al., 2024). Plasticity analyses were completed. Briefly, all data were converted to fold change scores (POST/PRE) and visualized for potential outliers (none were determined). Soft‐thresholding powers (β = 9 and 7 for serum and muscle, respectively) were determined through scale‐free topology (*R* ^2^ threshold of 0.8) using a ‘signed hybrid’ *networkType* (Supporting information, Figure S2). In the function *blockwiseModules* corType was set to ‘bicor’, and all remaining WGCNA settings were set as default. Modules were determined and assigned arbitrary colours which represent the eigenmetabolite (first principle component) for each module. Relatedness between serum metabolite modules and red cell volume, plasma ferritin, plasma sTfr, ${\dot{V}}_{O_{2}\mathrm{peak}}$ and TT performance were determined using module eigenmetabolite membership (MEM), a metric of relatedness between any metabolite and the eigenmetabolite. The same was completed for muscle metabolite modules and red cell volume, muscle Mgb, muscle Tfrc, muscle ferritin, ${\dot{V}}_{O_{2}\mathrm{peak}}$ and TT performance. Hub metabolites within each module were identified as having a MEM and metabolite trait significance (MTS) in the top 1/3 of values. If correlations were negative the MTS was evaluated for the bottom 1/3 of values. Lines matching the corresponding module colour on each figure depict the threshold for trait significance and module membership. Previously reported outcomes are presented as the mean ± SD fold change (FC, POST/PRE), unless stated otherwise.

## 3 RESULTS

### 3.1 Differential metabolites are more pronounced in muscle than serum

Of the 655 serum metabolites assessed, three metabolites increased (X‐11849, *N*‐acetylputrescine and cerotoylcarnitine; FDR ≤ 0.10) and one decreased (1‐margaroyl‐2‐arachidonoyl‐GPC (17:0/20:4); FDR ≤ 0.10) from PRE to POST (Supporting information, Table S1). Of the 495 muscle metabolites, 51 had an FDR ≤ 0.10 (Supporting information, Table S2). Serum and muscle metabolite differential data can be found in Supporting information, Tables S3 and S4.

### 3.2 Metabolomics modules

**FIGURE 2:**
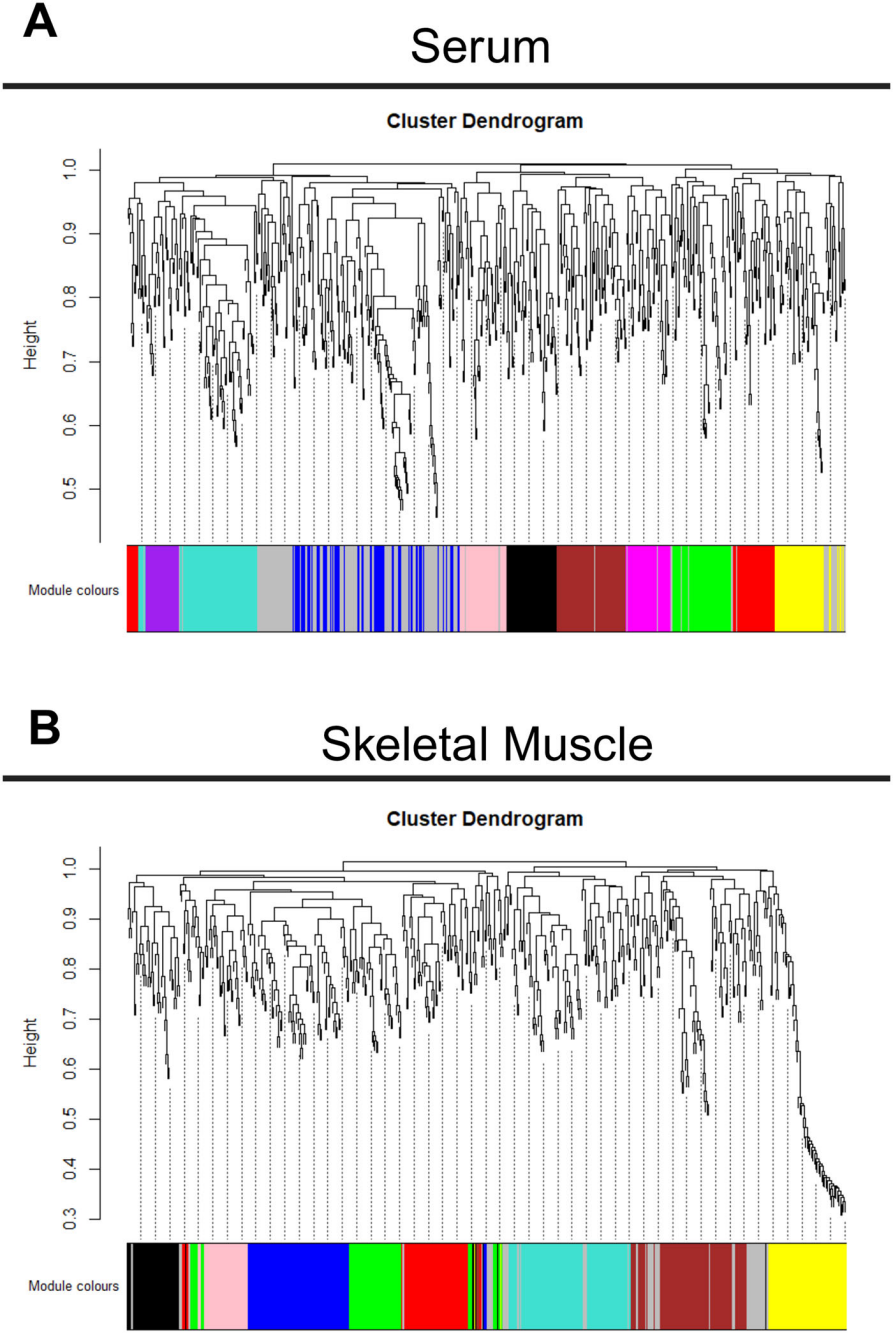
Cluster dendrogram based on dissimilarity demonstrating clustering of coexpressed metabolites and their module assignment for serum (a) and skeletal muscle (b). Branch height indicates the relatedness between metabolites using a biweight midcorrelation. Lowered colour bars represent the discrete module placement for metabolites.

Serum metabolites with significant associations were clustered into 11 discrete modules by WGCNA with colours representing the module eigenmetabolite (ME) (Figure 2a). Muscle metabolites with significant associations were clustered into nine discrete modules by WGCNA (Figure 2b). Serum and muscle metabolites not clustered to a module based on a lack of significance were assigned to their respective grey modules. These grey module metabolites do not possess a unique network structure and were deemed irrelevant for follow‐up correlative analyses. Previously published data used for associations with modules are represented in Table 1.

**TABLE 1:** Previously published outcomes.

	PRE (mean ± SD)	POST (mean ± SD)	Fold change (mean ± SD)	*P*
Red cell volume (mL)	2305.26 ± 324.24	2629.55 ± 397.04	1.14 ± 0.05	0.0005^*^
Time trial (s)	1804.63 ± 206.38	1638.88 ± 162.21	0.91 ± 0.07	0.01^*^
${\dot{V}}_{O_{2}\mathrm{peak}}$ (mL/kg/min)	44.13 ± 2.39	48.91 ± 3.47	1.11 ± 0.05	0.0003^*^
Plasma ferritin (ng mL^−1^)	46.99 ± 24.76	8.76 ± 4.14	0.19 ± 0.03	0.003^*^
Plasma sTfR (nM)	17.23 ± 1.05	35.67 ± 3.62	2.07 ± 0.18	<0.0001^*^
Muscle Mgb (AU)	1.00 ± 0.28	0.92 ± 0.23	0.94 ± 0.18	0.28
Muscle Tfrc (AU)	1.00 ± 0.64	1.32 ± 0.71	1.47 ± 0.56	0.02^*^
Muscle ferritin (AU)	1.00 ± 0.23	0.77 ± 0.28	0.75 ± 0.20	0.008^*^

Previously published outcomes (Drummer et al., 2024; Ryan et al., 2024).

* POST significantly different than PRE; *P* < 0.05.

### 3.3 Serum metabolomics modules are associated with improved TT performance

**FIGURE 3:**
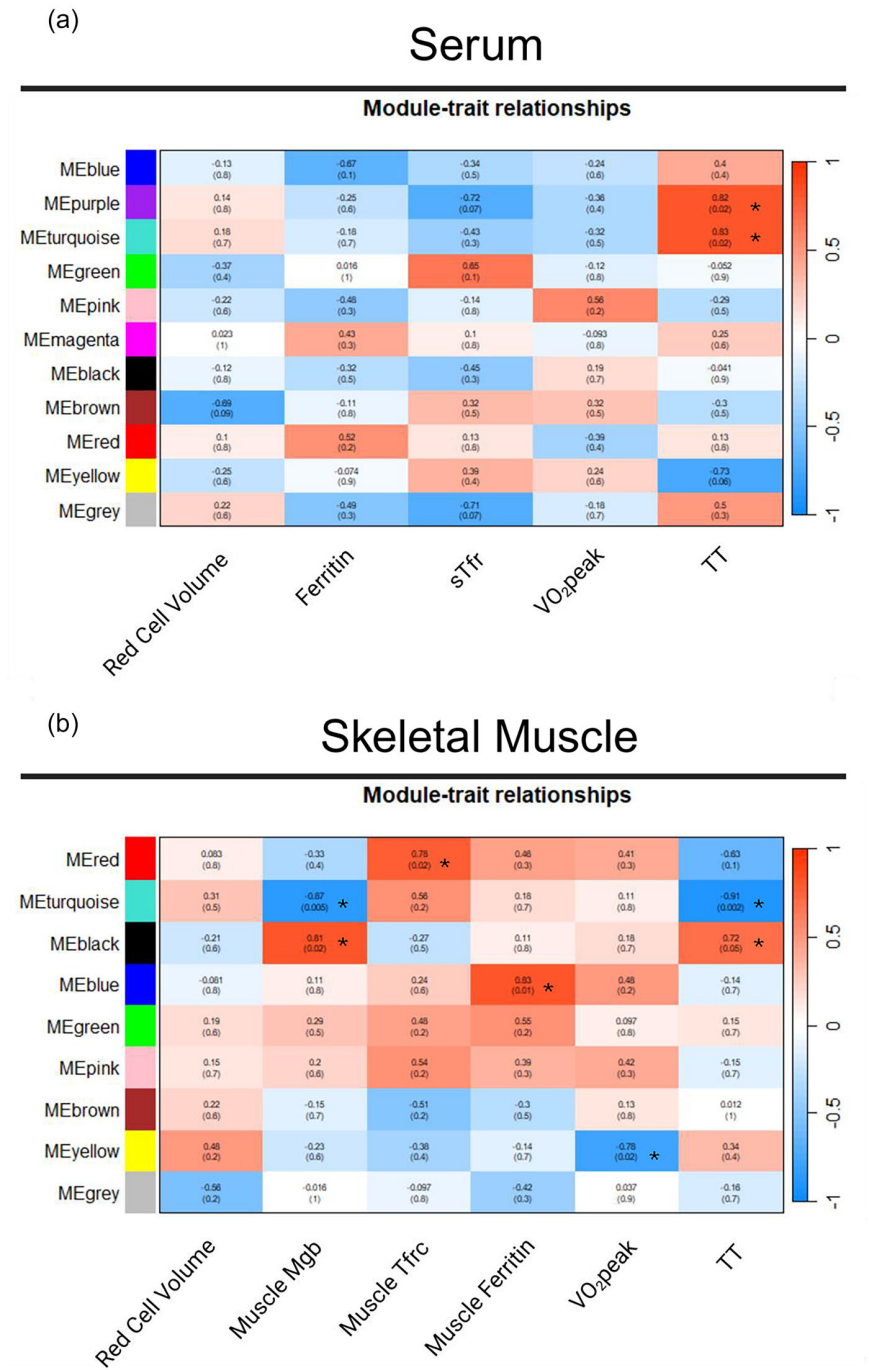
Serum (a) and muscle (b) module eigenmetabolites (ME) and variables of interest heat map used to determine significant modules for follow up interrogation. Values are represented as *R* and *P*‐value for each Pearson correlation. Significant modules are indicated by *.

**FIGURE 4:**
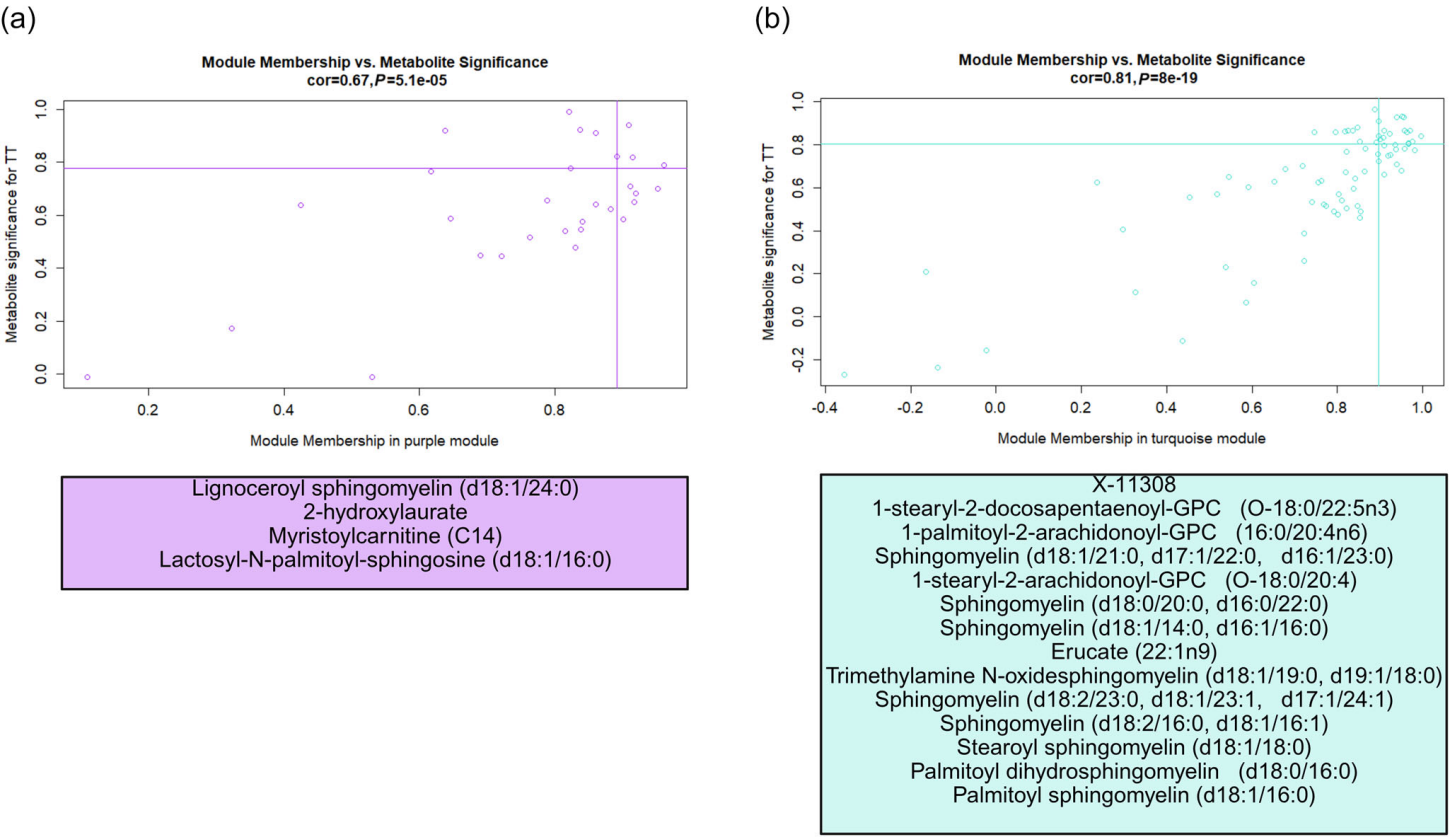
Serum module metabolites of interest plotted by trait significance with time trial (TT) and module membership for the purple module (a) and the turquoise module (b). Hub metabolites were identified as possessing trait significance and module membership values in the top 1/3 of values. Lines matching the corresponding module colour on each figure depict the threshold for trait significance and module membership. Hub metabolites reside in the upper right‐hand quadrant for positive correlations. These hub metabolites are displayed beneath each graph in the corresponding module colour.

Two serum eigenmetabolites (purple, *r* = 0.82, *P *= 0.02, and turquoise, *r* = 0.83, *P *= 0.02) were associated with changes in TT performance (Figure 3a). No modules were associated with red cell volume, ferritin, sTfr or ${\dot{V}}_{O_{2}\mathrm{peak}}$. The purple module contained 30 metabolites, while the turquoise module contained 76. The trait significance of metabolites in the purple module and module membership of those metabolites were correlated (*r* = 0.67 *P *= 5.1^−05^) (Figure 4a). Four of these metabolites were deemed hub metabolites as described in the methods (Figure 4a). The trait significance of metabolites in the turquoise module and module membership of those metabolites were correlated (*r* = 0.81 *P *= 8.0^−19^) (Figure 4b). Fourteen of these were deemed hub metabolites (Figure 4b).

### 3.4 Muscle metabolomics modules are associated with iron regulation and physical performance

**FIGURE 5:**
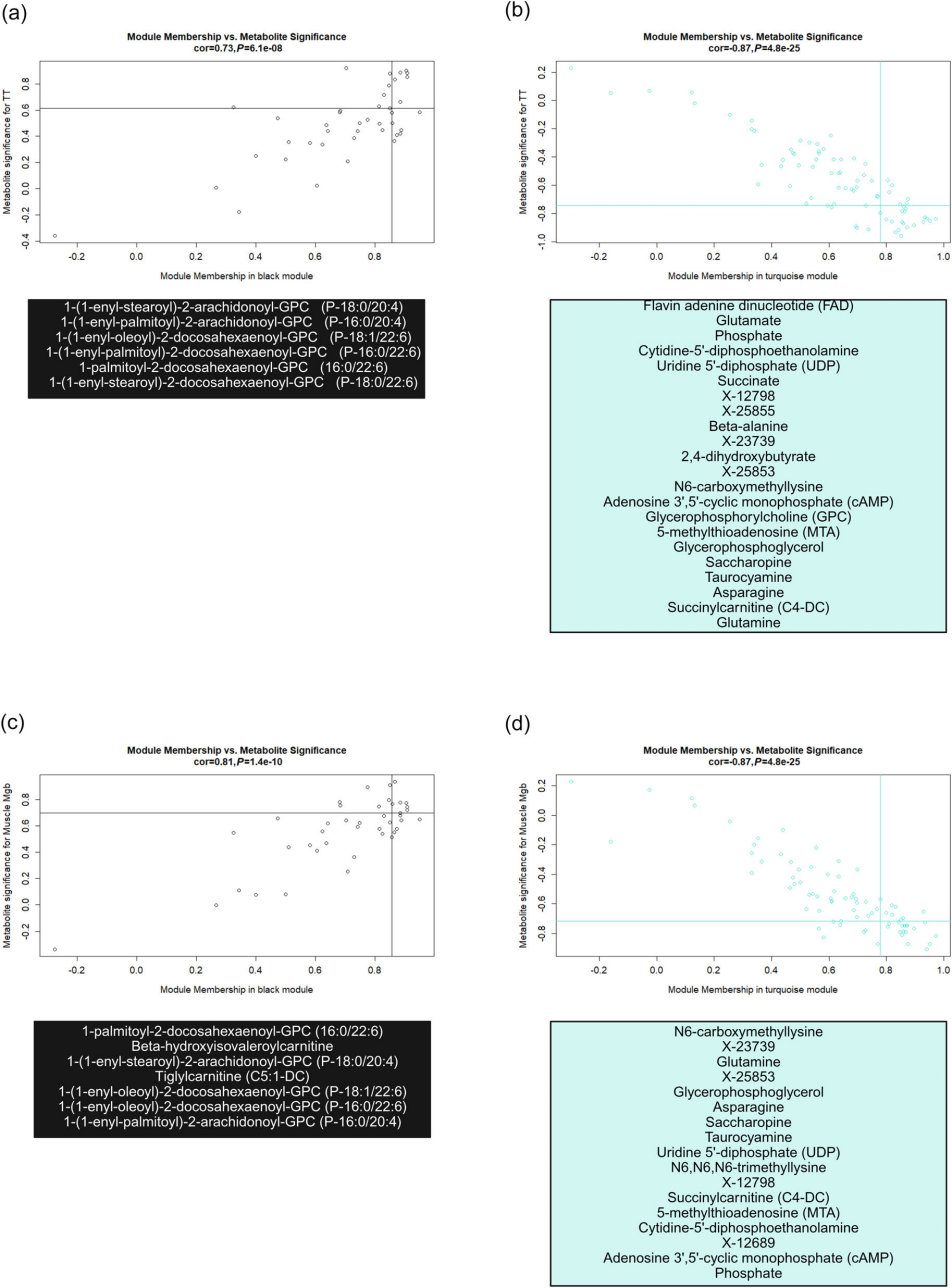
(a, b) Skeletal muscle module metabolites of interest plotted by trait significance with time trial (TT) and module membership for the black module (a) and the turquoise module (b). (c, d) Skeletal muscle module metabolites of interest plotted by trait significance with myoglobin (Mgb) and module membership for the black module (c) and the turquoise module (d). Hub metabolites were identified as possessing trait significance and module membership values in the top 1/3 of values. If correlations were negative, the trait significance was evaluated for the bottom 1/3 of values. Lines matching the corresponding module colour on each figure depict the threshold for trait significance and module membership. Hub metabolites reside in the upper right‐hand quadrant for positive correlations and lower right‐hand quadrant for negative correlations. These hub metabolites are displayed beneath each graph in the corresponding module colour.

**FIGURE 6:**
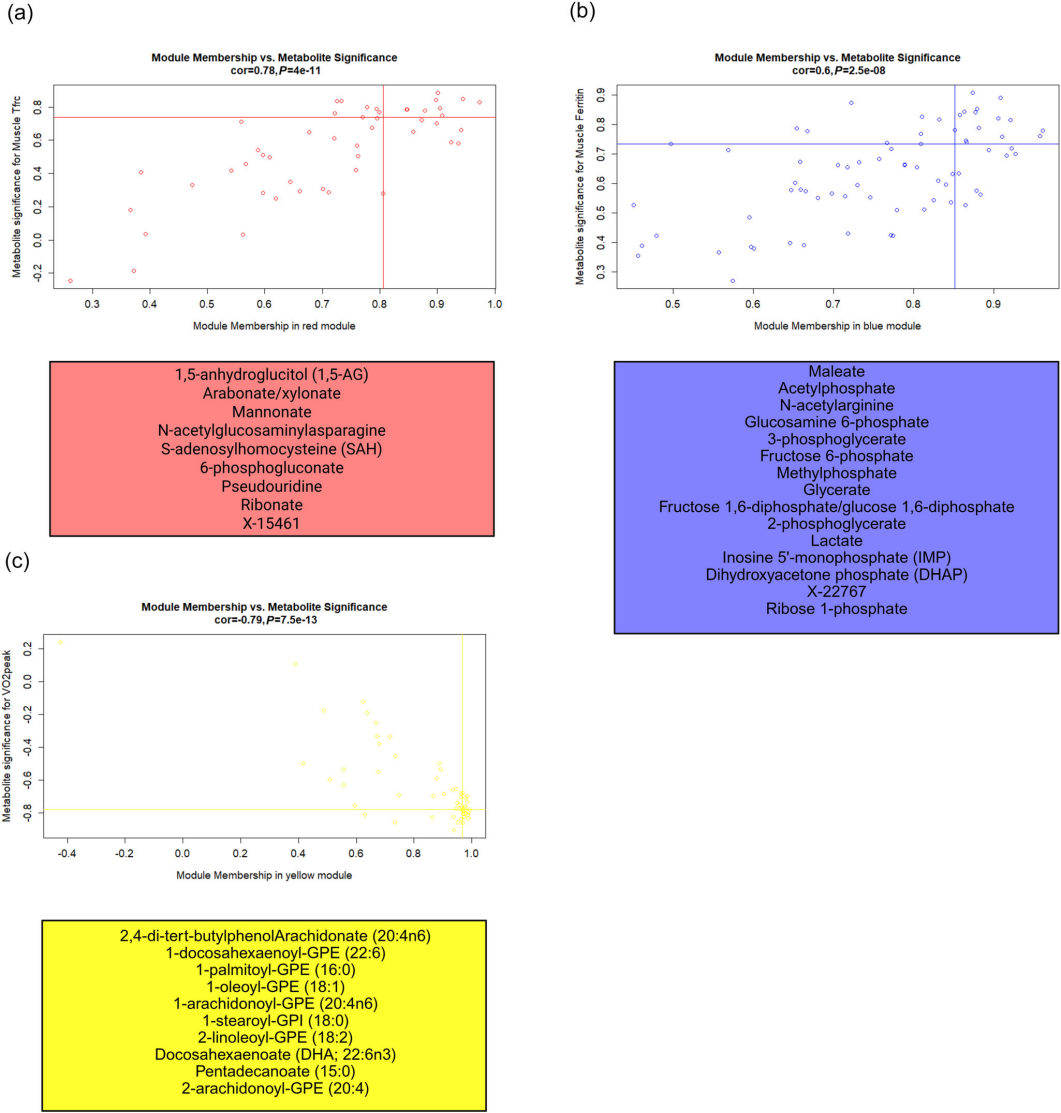
(a) Skeletal muscle module metabolites of interest plotted by trait significance with transferrin receptor (Tfrc) and module membership for the red module. (b) Skeletal muscle module metabolites of interest plotted by trait significance with muscle ferritin and module membership for the blue module. (c) Skeletal muscle module metabolites of interest plotted by trait significance with ${\dot{V}}_{O_{2}\mathrm{peak}}$ and module membership for the black module. Hub metabolites were identified as possessing trait significance and module membership values in the top 1/3 of values. If correlations were negative, the trait significance was evaluated for the bottom 1/3 of values. Lines matching the corresponding module colour on each figure depict the threshold for trait significance and module membership. Hub metabolites reside in the upper right‐hand quadrant for positive correlations and lower right‐hand quadrant for negative correlations. These hub metabolites are displayed beneath each graph in the corresponding module colour.

Seven muscle metabolomics modules were associated with either Mgb, or muscle Tfrc, or muscle ferritin, or ${\dot{V}}_{O_{2}\mathrm{peak}}$ and TT performance (Figure 3b). Two muscle eigenmetabolites (turquoise, *r* = −0.87, *P = *0.005, 78 metabolites, and black, *r* = 0.81, *P *= 0.02, 41 metabolites) were associated with muscle Mgb. The red (49 metabolites) and blue (72 metabolites) eigenmetabolites were positively associated with muscle Tfrc (*r* = 0.78, *P *= 0.02) and ferritin (*r* = 0.83, *P *= 0.01), respectively. The yellow eigenmetabolite was negatively associated with ${\dot{V}}_{O_{2}\mathrm{peak}}$ (*r* = −0.78, *P *= 0.02, 55 metabolites), and turquoise and black had opposing associations with TT performance (*r* = −0.91, *P *= 0.002, 78 metabolites, and *r* = 0.72, *P *= 0.05, 41 metabolites, respectively; as metabolites in black increase and metabolites in turquoise decrease, TT performance declines). No eigenmetabolites were associated with red cell volume. The association profiles for eigenmetabolites black and turquoise that correlated with TT and Mgb were mirrored, and their hub metabolites are represented in Figure 5a–d. The hub metabolites for red, blue and yellow associated with muscle Tfrc, ferritin and ${\dot{V}}_{O_{2}\mathrm{peak}}$ are represented in Figure 6a–c.

### 3.5 Overlapping metabolites in muscle and serum associated with TT

**FIGURE 7:**
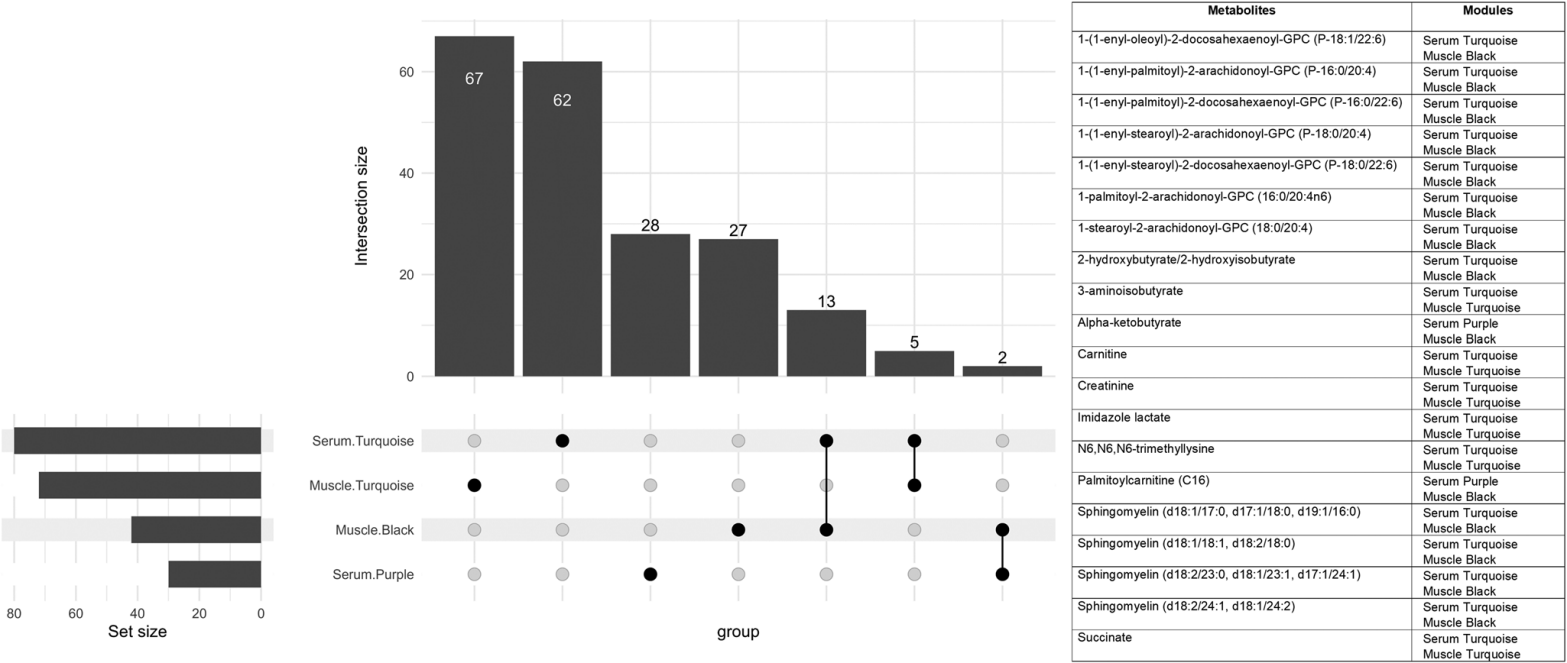
UpsetR plot (Conway et al., 2017) indicating shared overlap (intersection) of metabolites between modules associated with TT at the serum and muscle levels. Each metabolite with an intersection has been labelled with the corresponding modules it was grouped in.

Between muscle and serum metabolomics, four eigenmetabolites, serum turquoise, serum purple, muscle turquoise and muscle black, were associated with TT (Figure 3a, b). Three of these eigenmetabolites were positively associated with TT while muscle turquoise was negatively associated with TT. Of the 204 combined metabolites in these modules, 13 were shared between serum turquoise and muscle black, 5 were shared between serum turquoise and muscle turquoise, and 2 were shared between serum purple and muscle black (Figure 7).

## 4 DISCUSSION

The objective of this study was to examine changes in serum and skeletal muscle metabolomics and the associations of these metabolites with haematological and non‐haematological adaptations to strenuous exercise and EPO. The major finding was the skeletal muscle metabolome was more sensitive to 4 weeks of strenuous exercise and exogenous EPO administration than serum, with the altered metabolomes illustrating heighted fat oxidation and insulin sensitivity. Results from this study provide novel insight into changes in serum and skeletal muscle metabolomes after completing a strenuous training regimen coupled with EPO administration and suggest that these metabolic adaptations are associated with changes in markers of iron regulation and physical performance. Further, we have observed that skeletal muscle had a greater degree of change in metabolites compared with serum. Similarly, plasticity in skeletal muscle was far more robust than serum, with five modules associated (black, turquoise, red, blue, and yellow) with change in four traits of interest (TT, muscle Tfrc, muscle ferritin and ${\dot{V}}_{O_{2}\mathrm{peak}}$) compared to serum with two modules (purple and turquoise) associated with change in TT.

Three of the four significantly different serum metabolites were annotated to polyamine metabolism, phosphatidylcholine and fatty acid metabolism (acyl carnitine, long chain saturated) pathways. Polyamines can regulate oxidative stress and autophagy, although primarily illustrated in model systems such as mice (Galasso et al., 2023; Sagar et al., 2021). The differential polyamine metabolites may be due to the exercise and EPO intervention, as exercise training results in increased autophagic flux (Botella et al., 2022), and autophagy aids in the formation of mature red blood cells (Grosso et al., 2017). Serum cerotoylcarnitine was higher at POST and is part of the fatty acid metabolism (acyl carnitine, long chain saturated) pathway. This supports results from the parent study (Drummer et al., 2024) and aligns with the work of others (Caillaud et al., 2015; Christensen et al., 2012; Larsen et al., 2021) where increased fat oxidation or metabolites in lipid metabolism (Lima et al., 2023) has been observed after EPO administration. Additionally, our previous work found that acyl carnitines were increased after military training consisting of strenuous aerobic exercise (Karl et al., 2017). The fourth significant metabolite was X‐11849, an unannotated metabolite which may be identified in the future as technologies continue to improve (Sana et al., 2013).

Differential skeletal muscle metabolites were primarily annotated to the phosphatidylcholine (PC), plasmalogen and sphingomyelin pathways. Human sarcoplasmic reticulum is mostly made up of the phospholipids, with PC contributing to approximately 65% of the membrane (Takagi, 1971). Increases in PC in the current study are in agreement with previous work which reported 12 weeks of concurrent exercise training increases PC within skeletal muscle (Lee et al., 2018). Similarly, endurance trained athletes have been reported to have higher PC than sedentary individuals with obesity and individuals with type 2 diabetes (Newsom et al., 2016). As the constitution of skeletal muscle membrane phospholipids impact the integrity and function of the cellular membrane, changes in PC have been suggested to impact membrane trafficking, improving insulin sensitivity and mitochondrial function (Grapentine & Bakovic, 2019). Indeed, Clore et al. (1998) reported measurements of insulin sensitivity using a hyperinsulinaemic euglycaemic clamp technique in men and women was significantly associated with PC composition within skeletal muscle. Concurring with this past work, following exercise and EPO, the parent study reported greater glucose metabolic clearance rate, a marker of insulin sensitivity, from PRE to POST during 90 min of steady state exercise (Drummer et al., 2024). The observed remodelling of the lipid pool in this work, accompanied with the greater metabolic clearance rate in the parent trial, may indicate greater insulin sensitivity. However, it should be noted that as no direct measurement of insulin sensitivity was conducted in the current study and our sample size was limited, links to PC and insulin sensitivity in the current study should be taken as speculative and hypothesis‐generating, thus needing further direct interrogation.

Using WGCNA, we identified two serum modules, purple and turquoise, with positive associations with TT performance. Many of the hub metabolites within these modules are sphingomyelins. Sphingomyelins are found in cell membranes and make up the greatest proportion of sphingolipids (McCluskey et al., 2022; Tan‐Chen et al., 2020). Additionally, they function in membrane fluidity and act as signalling molecules. A randomized control trial comparing the effects of combined aerobic and resistance training vs. control on mitochondrial respiration and phospholipid signatures in obese females found the control group contained a higher proportion of sphingomyelins following the 12‐week intervention compared to those who underwent the exercise intervention (Mendham et al., 2021). These findings coupled with work demonstrating that higher sphingomyelins are associated with greater incidence of cardiovascular disease (Kikas et al., 2018) suggest that they may be an indicator of cardiorespiratory fitness. This aligns with the present observations, as sphingomyelins increased as time to complete the TT lengthened.

Five muscle modules were associated with iron regulation and performance outcomes. Among these, the turquoise module was negatively associated with TT performance, and in the hub metabolites, β‐alanine was found. β‐Alanine is the rate limiting factor in carnosine production, and dietary supplementation has been shown to increase muscle carnosine levels (Culbertson et al., 2010). β‐Alanine seemingly exerts its effect by increasing carnosine and buffering pH (Gonzalez et al., 2022). The negative association may reflect an association between buffering capacity and performance. When looking at the change in β‐alanine in our differential metabolite analyses, this appears to hold true. While there was a decrease in β‐alanine from PRE to POST, the volunteers that maintained the highest muscle β‐alanine content had the greatest improvement in TT performance. However, these data are associative and hypothesis‐generating, thus presenting the opportunity for future efforts to determine causation.

Both the serum and muscle modules that included carnitine were associated with change in TT performance. However, the direction of association was opposing. The TT performance improved as carnitine increased in muscle and decreased in serum. There is a precedent for metabolites differing between muscle and blood since muscle is highly metabolically active while blood is considered metabolically moderate (Kiseleva et al., 2021; Xu et al., 2016). Further, blood collects metabolites from all tissues, presenting a non‐specific global snapshot at a specific time point (Kiseleva et al., 2021). Specific to data from the present study, muscle cannot synthesize carnitine and must uptake it from circulation (Flanagan et al., 2010; Furuichi et al., 2023). Carnitine facilitates fatty acid transport across mitochondrial membranes for oxidation (Furuichi et al., 2023; Gnoni et al., 2020). The positive association between carnitine and aerobic performance coupled with enhanced fat oxidation observed in the parent trial is consistent with changes driving altered fuel utilization that occurred PRE to POST.

Within skeletal muscle, the blue module was positively associated with muscle ferritin, and many hub metabolites were part of carbohydrate metabolism (glucosamine 6‐phosphate, 3‐phosphoglycerate, fructose 6‐phosphate, glycerate, etc.). Since muscle ferritin was lower at POST than PRE, it suggests that a lower abundance in metabolites involved in carbohydrate metabolism is associated with low muscle ferritin possibly due to the heightened reliance on oxidative metabolism. In our previous work, we observed that the upregulation of erythropoiesis from strenuous exercise and EPO administration led to a reduction of muscle ferritin indicating an alteration in tissue iron regulation (Ryan et al., 2024). Coupled with the increase in muscle transferrin receptor, strenuous training with EPO led to decreased muscle iron storage to prioritize red blood cell production despite a diet robust in iron (Ryan et al., 2024; Torti & Torti, 2002). Further, the parent study observed enhanced fat oxidation and transcription of mitochondrial activity markers (Drummer et al., 2024). Iron is a necessary cofactor within the mitochondrial respiratory complexes (Yiannikourides & Latunde‐Dada, 2019). Overall, these metabolites may be capturing increased fat oxidation or metabolic changes in muscle due to the altered demand for iron.

The current study provides novel exploratory insights into the effects of prolonged strenuous training and EPO administration on serum and skeletal muscle metabolomes. However, this work is not without limitations. It must be noted that without exercise and EPO‐only groups, the current study is unable to delineate EPO‐specific effects on observed changes in circulating and skeletal muscle metabolites. While the data generated from this study provide information that will facilitate the generation of hypotheses for future studies, outcomes of the current work cannot prove specific mechanistic actions. Furthermore, the imputation of missing data for the metabolomics analysis should also be considered. Imputation of missing values is common in large metabolomics datasets (Do et al., 2018); however, given the relatively small sample size of our current study, potential bias in the analysis should be considered when imputing missing values. This again suggests that future mechanistic studies are required to confirm results presented in the current study. Additionally, participants were young healthy males, and metabolomic signatures differ by sex, age and disease state (Aderemi et al., 2021; Costanzo et al., 2022). While females were not excluded from this work, only males consented to participate. Additional studies should be conducted in female participants and in both sexes across the age and disease spectrum to determine if these observations can be generalized outside of healthy males. Further, the sample size was small due to the parent trial being powered for primary outcomes (Drummer et al., 2024); however, conservative approaches were taken to account for this within the WGCNA. Additionally, other work in mice (Zhou et al., 2024) and humans (Vernocchi et al., 2020) has used similar sample sizes. These conservative approaches paired with the metabolomics data mechanistically aligning with the outcomes in the parent trial provide additional confidence in these observations. While the single metabolite data support the heighted fat oxidation and insulin sensitivity at rest, this was not directly assessed and therefore cannot be confirmed. However, greater fat oxidation has consistently been observed following EPO administration, and metabolite clearance rates were higher throughout exercise at POST compared to PRE during the parent trial. Despite this conservative approach, the lack of a placebo group and limited sample size precludes causal inference and necessitates cautious interpretation.

In conclusion, the skeletal muscle metabolome was more sensitive to 4 weeks of strenuous exercise and exogenous EPO administration than serum. These altered metabolomes provide insight into possible underlying mechanisms contributing to the heightened fat oxidation and insulin sensitivity observed in the parent trial. Further plasticity analyses with WGCNA revealed metabolite networks associated with performance and iron homeostasis outcomes in serum and skeletal muscle. Greater retention of hub metabolites like β‐alanine were associated with improved TT performance. Additionally, hub metabolites involved in carbohydrate metabolism were reduced and may depict increased fat oxidation or metabolic changes in muscle due to the demand for iron. These observations provide insight on potential metabolic underpinnings driving improved physical performance, heightened fat oxidation and altered iron regulation after 28 days of strenuous exercise and EPO administration.

## AUTHOR CONTRIBUTIONS

Lee M. Margolis, Stefan M. Pasiakos, James P. McClung, Benjamin J. Ryan, and Devin J. Drummer designed research; Devin J. Drummer, Julie L. McNiff, Emily E. Howard, Jess A. Gwin, Christopher T. Carrigan, Nancy E. Murphy, Marques A. Wilson, David E. Barney Jr, Benjamin J. Ryan, and Lee M. Margolis performed research; Devin J. Drummer and Lee M. Margolis analysed data; Devin J. Drummer and Lee M. Margolis interpreted results; Devin J. Drummer and Lee M. Margolis prepared tables and figures; Devin J. Drummer and Lee M. Margolis drafted manuscript. All authors have read and approved the final version of this manuscript and agree to be accountable for all aspects of the work in ensuring that questions related to the accuracy or integrity of any part of the work are appropriately investigated and resolved. All persons designated as authors qualify for authorship, and all those who qualify for authorship are listed.

## CONFLICT OF INTEREST

None declared.

## Supporting information



Supporting Information

Supporting Information

Supporting Information

Supporting Information

Supporting Information

Supporting Information

## Data Availability

Data are available upon request and approval of a data sharing agreement from the corresponding author, Lee Margolis, lee.m.margolis.civ@health.mil. Supplemental Tables and Figure: (Private link for review will be made public upon acceptance: https://figshare.com/s/95563648e841b030c8fa).
