# Supplementary material for: Metabolomic responses are more sensitive in muscle than serum following 28 days of arduous exercise with erythropoietin administration
Source: Exp Physiol. 2026 Apr 11;111(5):2613–26. doi: 10.1113/EP093342 (PMC13131109; doi:10.1113/EP093342)
Supplement: Supplementary file 1 — Supporting Information [file EPH-111-2613-s002.pdf]

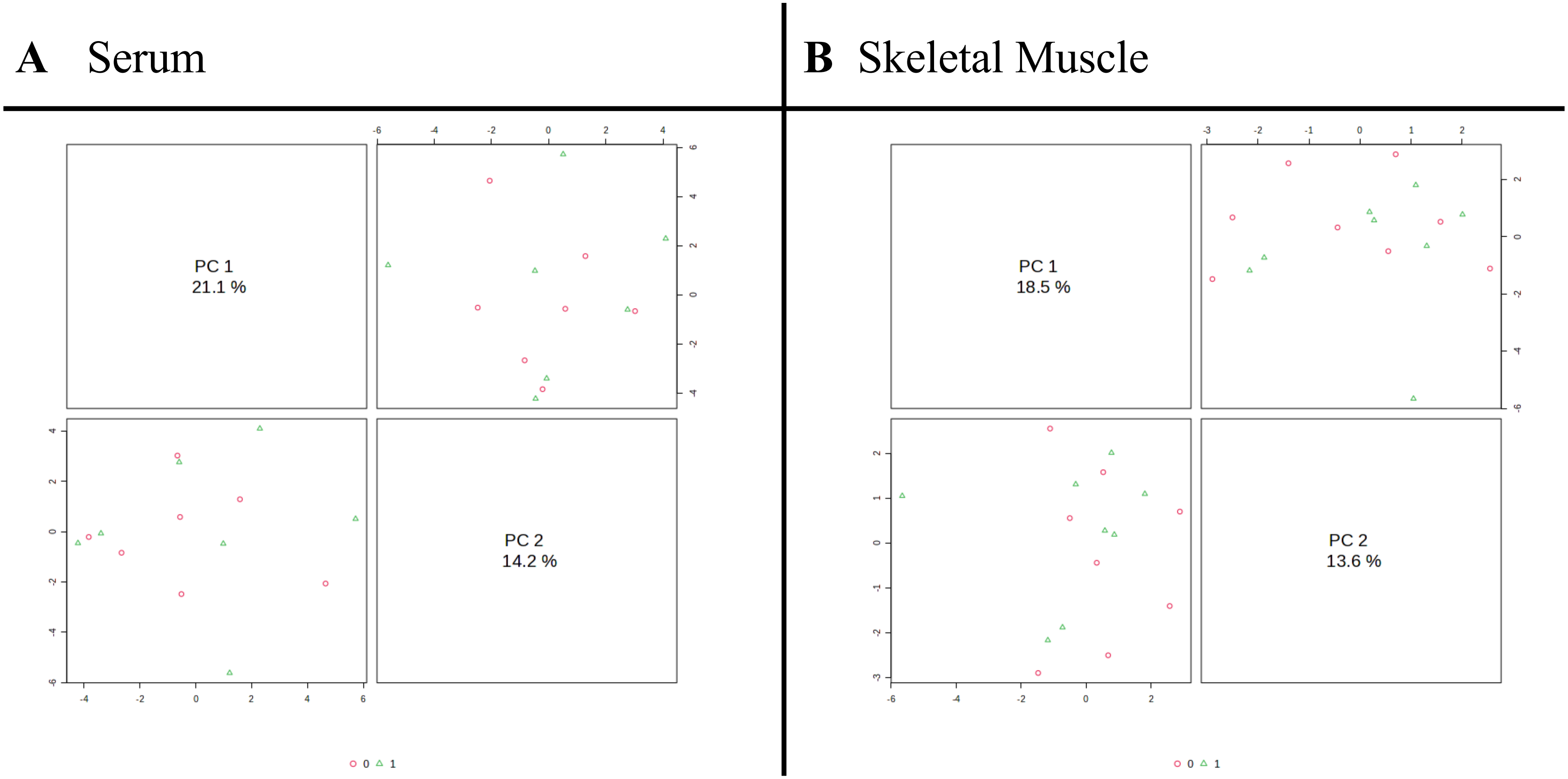

Supplementary Figure 1. Principal Component Analyses for serum A), and skeletal muscle B). ○ = PRE, △ = POST.
