# Supplementary material for: Metabolomic responses are more sensitive in muscle than serum following 28 days of arduous exercise with erythropoietin administration
Source: Exp Physiol. 2026 Apr 11;111(5):2613–26. doi: 10.1113/EP093342 (PMC13131109; doi:10.1113/EP093342)
Supplement: Supplementary file 2 — Supporting Information [file EPH-111-2613-s001.pdf]

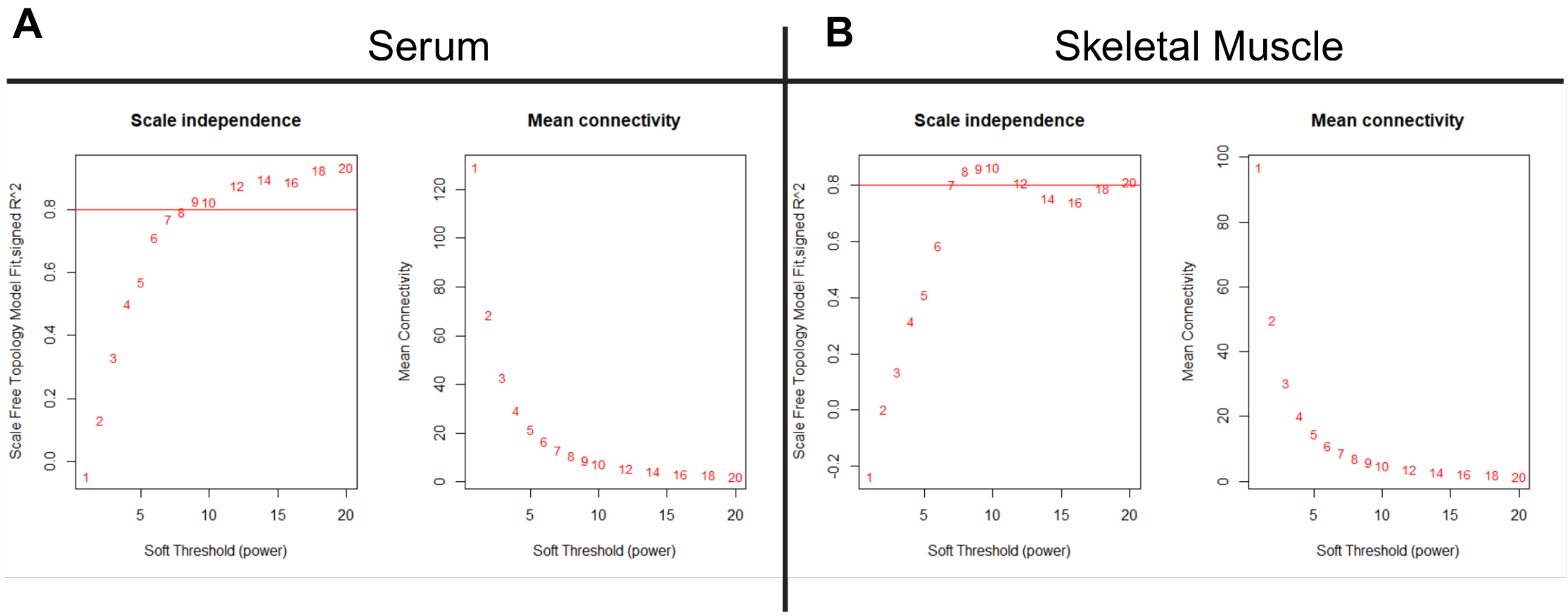

Supplementary Figure 2. Scale free network topology plot fitting and mean connectivity against soft-thresholding power for serum **A)** and skeletal muscle **B)**. This was utilized to determine soft-thresholding power for subsequent WGCNA functions (e.g., network construction, topological overlap matrix).
