# Supplementary material for: Metabolomic responses are more sensitive in muscle than serum following 28 days of arduous exercise with erythropoietin administration
Source: Exp Physiol. 2026 Apr 11;111(5):2613–26. doi: 10.1113/EP093342 (PMC13131109; doi:10.1113/EP093342)
Supplement: Supplementary file 3 — Supporting Information [file EPH-111-2613-s006.docx]

Table 1. Differentially Expressed Serum Metabolites

| Serum Metabolites | *P*-Value | FDR | Average % Change | Average % Change SD | Sub-pathway Annotation from Metabolon |
| --- | --- | --- | --- | --- | --- |
| N-acetylputrescine | 0.00 | 0.10 | 1.28 | 0.48 | Polyamine Metabolism |
| X-11849 | 0.00 | 0.10 | 18.30 | 8.98 |  |
| 1-margaroyl-2-arachidonoyl-GPC (17:0/20:4) | 0.00 | 0.10 | -0.92 | 0.35 | Phosphatidylcholine |
| Cerotoylcarnitine (C26) | 0.00 | 0.10 | 3.05 | 1.26 | Fatty Acid Metabolism |

Comparison POST vs PRE; paired Ttests (*P*-Value) corrected with Benjamini-Hochberg False Discovery Rate (FDR). Average percent change = (POST-PRE)/PRE*100
