# Supplementary material for: Metabolomic responses are more sensitive in muscle than serum following 28 days of arduous exercise with erythropoietin administration
Source: Exp Physiol. 2026 Apr 11;111(5):2613–26. doi: 10.1113/EP093342 (PMC13131109; doi:10.1113/EP093342)
Supplement: Supplementary file 4 — Supporting Information [file EPH-111-2613-s004.docx]

Table 2. Differentially Expressed Muscle Metabolites

| Muscle Metabolites | P-Value | FDR | Average % Change | Average % Change SD | Sub-pathway Annotation from Metabolon |
| --- | --- | --- | --- | --- | --- |
| N,N,N-trimethyl-alanylproline betaine (TMAP) | 0.00 | 0.00 | -0.47 | 0.11 | Urea cycle; Arginine and Proline Metabolism |
| Deoxycarnitine | 0.00 | 0.01 | -1.39 | 0.35 | Carnitine Metabolism |
| 3-hydroxystachydrine | 0.00 | 0.01 | -7.97 | 2.01 | Food Component/Plant |
| 1-stearoyl-2-arachidonoyl-GPI (18:0/20:4) | 0.00 | 0.01 | -0.53 | 0.19 | Phosphatidylinositol (PI) |
| 1-(1-enyl-palmitoyl)-2-docosahexaenoyl-GPC (P-16:0/22:6) | 0.00 | 0.01 | -3.25 | 1.28 | Plasmalogen |
| Choline phosphate | 0.00 | 0.01 | 2.23 | 0.60 | Phospholipid Metabolism |
| 1-(1-enyl-palmitoyl)-2-arachidonoyl-GPC (P-16:0/20:4) | 0.00 | 0.02 | -2.65 | 1.15 | Plasmalogen |
| 1-palmitoyl-2-eicosapentaenoyl-GPE (16:0/20:5) | 0.00 | 0.02 | 2.29 | 0.96 | Phosphatidylethanolamine (PE) |
| 1-palmitoyl-2-linoleoyl-GPE (16:0/18:2) | 0.00 | 0.02 | 1.74 | 0.65 | Phosphatidylethanolamine (PE) |
| 1-(1-enyl-stearoyl)-2-arachidonoyl-GPE (P-18:0/20:4) | 0.00 | 0.03 | -1.47 | 0.75 | Plasmalogen |
| 1-(1-enyl-oleoyl)-2-docosahexaenoyl-GPC (P-18:1/22:6) | 0.00 | 0.03 | -3.75 | 1.97 | Plasmalogen |
| X-25419 | 0.00 | 0.03 | -1.33 | 0.66 |  |
| 1-oleoyl-2-linoleoyl-GPC (18:1/18:2) | 0.00 | 0.03 | 1.29 | 0.72 | Phosphatidylcholine (PC) |
| Hydroxy-N6,N6,N6-trimethyllysine | 0.00 | 0.03 | -2.67 | 1.06 | Lysine Metabolism |
| 1-margaroyl-2-linoleoyl-GPC (17:0/18:2) | 0.00 | 0.03 | 0.64 | 0.23 | Phosphatidylcholine (PC) |
| N-acetyl-aspartyl-glutamate (NAAG) | 0.00 | 0.03 | -2.33 | 1.25 | Glutamate Metabolism |
| 1-linoleoyl-2-arachidonoyl-GPC (18:2/20:4n6) | 0.00 | 0.03 | 1.32 | 0.77 | Phosphatidylcholine (PC) |
| Myo-inositol | 0.00 | 0.04 | -2.75 | 1.26 | Inositol Metabolism |
| X-23593 | 0.00 | 0.04 | -0.97 | 0.60 |  |
| 1-(1-enyl-stearoyl)-2-docosahexaenoyl-GPC (P-18:0/22:6) | 0.00 | 0.04 | -3.24 | 2.02 | Plasmalogen |
| Homostachydrine | 0.00 | 0.05 | 3.52 | 2.30 | Food Component/Plant |
| 1,2-dilinoleoyl-GPC (18:2/18:2) | 0.00 | 0.05 | 1.55 | 0.97 | Phosphatidylcholine (PC) |
| 1-(1-enyl-palmitoyl)-2-arachidonoyl-GPE (P-16:0/20:4) | 0.00 | 0.05 | -0.68 | 0.45 | Plasmalogen |
| Cytidine 5'-diphosphocholine | 0.00 | 0.05 | -4.34 | 2.28 | Phospholipid Metabolism |
| Phosphate | 0.00 | 0.06 | -0.35 | 0.25 | Oxidative Phosphorylation |
| 1-(1-enyl-stearoyl)-2-arachidonoyl-GPC (P-18:0/20:4) | 0.00 | 0.06 | -3.17 | 2.23 | Plasmalogen |
| N,N-dimethyl-pro-pro | 0.00 | 0.06 | -0.75 | 0.53 | Modified Peptides |
| X-11381 | 0.00 | 0.06 | -0.63 | 0.45 |  |
| Sphingomyelin (d18:1/19:0, d19:1/18:0) | 0.00 | 0.06 | -2.84 | 1.66 | Sphingomyelins |
| N-acetylthreonine | 0.00 | 0.06 | -1.78 | 1.27 | Glycine, Serine and Threonine Metabolism |
| Carnitine | 0.00 | 0.07 | -0.25 | 0.19 | Carnitine Metabolism |
| 1-(1-enyl-stearoyl)-2-oleoyl-GPE (P-18:0/18:1) | 0.00 | 0.07 | -1.82 | 1.00 | Plasmalogen |
| X-12015 | 0.01 | 0.09 | -3.29 | 2.52 |  |
| Tricosanoyl sphingomyelin (d18:1/23:0) | 0.01 | 0.09 | -0.93 | 0.76 | Sphingomyelins |
| Adenosine 5'-diphosphate (ADP) | 0.01 | 0.09 | -1.66 | 0.96 | Purine Metabolism, Adenine containing |
| Stearoyl sphingomyelin (d18:1/18:0) | 0.01 | 0.09 | -1.06 | 0.88 | Sphingomyelins |
| 1-palmitoyl-2-dihomo-linolenoyl-GPC (16:0/20:3n3 or 6) | 0.01 | 0.09 | 0.58 | 0.40 | Phosphatidylcholine (PC) |
| Sphingadienine | 0.01 | 0.09 | 3.85 | 2.19 | Sphingolipid Synthesis |
| Lignoceroyl sphingomyelin (d18:1/24:0) | 0.01 | 0.09 | -1.20 | 1.01 | Sphingomyelins |
| Kynurenine | 0.01 | 0.09 | 3.30 | 2.09 | Tryptophan Metabolism |
| 1-pentadecanoyl-2-linoleoyl-GPC (15:0/18:2) | 0.01 | 0.09 | 1.05 | 0.85 | Phosphatidylcholine (PC) |
| 2-piperidinone | 0.01 | 0.09 | 5.12 | 4.25 | Food Component/Plant |
| Behenoyl sphingomyelin (d18:1/22:0) | 0.01 | 0.09 | -1.05 | 0.86 | Sphingomyelins |
| Methionine | 0.01 | 0.09 | -1.00 | 0.87 | Methionine, Cysteine, SAM and Taurine Metabolism |
| X-24801 | 0.01 | 0.09 | -1.20 | 1.04 |  |
| 1-linoleoyl-2-linolenoyl-GPC (18:2/18:3) | 0.01 | 0.09 | 2.80 | 2.26 | Phosphatidylcholine (PC) |
| 1-(1-enyl-stearoyl)-2-docosapentaenoyl-GPE (P-18:0/22:5n3) | 0.01 | 0.09 | -1.22 | 1.01 | Plasmalogen |
| 3-(4-hydroxyphenyl)lactate | 0.01 | 0.10 | 1.06 | 0.95 | Tyrosine Metabolism |
| Sphingomyelin (d18:1/18:1, d18:2/18:0) | 0.01 | 0.10 | -0.78 | 0.69 | Sphingomyelins |
| Glycerophosphorylcholine (GPC) | 0.01 | 0.10 | -1.03 | 0.87 | Phospholipid Metabolism |
| 1-linoleoyl-2-arachidonoyl-GPE (18:2/20:4) | 0.01 | 0.10 | 12.39 | 11.15 | Phosphatidylethanolamine (PE) |

Comparison POST vs PRE; paired Ttests (*P*-Value) corrected with Benjamini-Hochberg False Discovery Rate (FDR). Average percent change = (POST-PRE)/PRE*100
